# Abnormal Resting-State Functional Connectivity in Progressive Supranuclear Palsy and Corticobasal Syndrome

**DOI:** 10.3389/fneur.2017.00248

**Published:** 2017-06-06

**Authors:** Komal Bharti, Matteo Bologna, Neeraj Upadhyay, Maria Cristina Piattella, Antonio Suppa, Nikolaos Petsas, Costanza Giannì, Francesca Tona, Alfredo Berardelli, Patrizia Pantano

**Affiliations:** ^1^Department of Neurology and Psychiatry, Sapienza University of Rome, Rome, Italy; ^2^Neuromed Institute IRCCS, Pozzilli, Isernia, Italy

**Keywords:** resting-state functional magnetic resonance imaging, resting-state networks, functional connectivity, progressive supranuclear palsy, corticobasal syndrome

## Abstract

**Background:**

Pathological and MRI-based evidence suggests that multiple brain structures are likely to be involved in functional disconnection between brain areas. Few studies have investigated resting-state functional connectivity (rsFC) in progressive supranuclear palsy (PSP) and corticobasal syndrome (CBS). In this study, we investigated within- and between-network rsFC abnormalities in these two conditions.

**Methods:**

Twenty patients with PSP, 11 patients with CBS, and 16 healthy subjects (HS) underwent a resting-state fMRI study. Resting-state networks (RSNs) were extracted to evaluate within- and between-network rsFC using the Melodic and FSLNets software packages.

**Results:**

Increased within-network rsFC was observed in both PSP and CBS patients, with a larger number of RSNs being involved in CBS. Within-network cerebellar rsFC positively correlated with mini-mental state examination scores in patients with PSP. Compared to healthy volunteers, PSP and CBS patients exhibit reduced functional connectivity between the lateral visual and auditory RSNs, with PSP patients additionally showing lower functional connectivity between the cerebellar and insular RSNs. Moreover, rsFC between the salience and executive-control RSNs was increased in patients with CBS compared to HS.

**Conclusion:**

This study provides evidence of functional brain reorganization in both PSP and CBS. Increased within-network rsFC could represent a higher degree of synchronization in damaged brain areas, while between-network rsFC abnormalities may mainly reflect degeneration of long-range white matter fibers.

## Introduction

Progressive supranuclear palsy (PSP) and corticobasal syndrome (CBS) are neurodegenerative disorders characterized by parkinsonism among other clinical features (1, 2). Both PSP and CBS share tau protein aggregation and deposition, which leads to neurodegeneration in a number of brain areas (3).

MRI studies have revealed gray matter abnormalities at multiple brain levels in both PSP and CBS: subcortical nuclei pathology is involved in both conditions, but PSP patients exhibit predominant midbrain atrophy and a limited involvement of frontal cortex pathology, while CBS patients display more evident cortical atrophy, particularly in peri-rolandic areas (4–7). Moreover, diffusion tensor imaging has revealed widespread white matter bundle damage, which suggests altered structural connectivity between cortical and subcortical structures, in both PSP and CBS patients (4–8).

Resting-state functional magnetic resonance imaging (rsfMRI) is an advanced technique able to monitor oscillations in the blood oxygen level dependent (BOLD) signal across time, under non-arousal (resting) states. The temporal correlation of these BOLD signal fluctuations across various brain regions may reflect a resting-state functional connectivity (rsFC) between them, i.e., the presence of resting-state network (RSN) (9). Few rsfMRI studies on PSP patients adopted a seed-based analysis, which requires an *a priori* hypothesis, and showed disrupted rsFC in several subcortical nuclei (10–12). By contrast, only one study on CBS patients showed rsFC changes in the sensorimotor network that was associated with motor and cognitive abnormalities (13).

Independent component analysis (ICA) can be used to analyze whole-brain rsFC and synchronous spontaneous activity to determine well-defined large-scale RSNs without *a priori* hypotheses (14). ICA has previously been used to study rsFC in neurodegenerative diseases (15–17). To the best of our knowledge, the ICA approach has not yet been adopted to investigate rsFC in patients with PSP and CBS. The ICA analysis may provide an important insight into the global functional reorganization of the brain following gray and white matter neurodegeneration in both conditions.

Since tau pathology affects various brain areas in PSP and CBS (3–6), our hypothesis is that structural damage may differently affect functional connectivity in these two disorders. Therefore, we investigated within- and between-network rsFC abnormalities in PSP and CBS patients. We also investigated possible relationships between rsfMRI data and clinical features of patients.

## Materials and Methods

### Participants

Twenty patients with clinically probable PSP and 11 patients with clinically probable CBS were enrolled in the study. Sixteen age- and sex-matched healthy subjects (HS) with no history of neurological or psychiatric illness were enrolled as a control group. All participants had no clinical history or evidences on standard MRI sequences of vascular diseases. From the standard MRI sequences, there were no evidences of hippocampal atrophy in any participants. All the participants were recruited at the Department of Neurology and Psychiatry, Sapienza University of Rome, Italy by neurologists experienced in Movement Disorders. The diagnosis of PSP and CBS was based on clinical criteria (2, 18). The clinical assessment included the Unified Parkinson’s Disease Rating Scale (UPDRS) part III (19), Hoehn and Yahr scale (H&Y) (20), Mini-Mental State Evaluation (MMSE) (21), and Frontal Assessment Battery (FAB) (22). Participants gave their written informed consent to the study, which was approved by the institutional review board and conformed to the declaration of Helsinki.

### Imaging Protocol

A multimodal MRI study was performed on all the participants according to a standardized protocol on a 3-T scanner (Magnetic Verio; Siemens, Erlangen, Germany) using a 12-channel head coil designed for parallel imaging (generalized autocalibrating partially parallel acquisitions). Before rsfMRI acquisition, participants were instructed to lie down, awake, and with their eyes closed, in a fully relaxed condition. The rsfMRI study included 140 volumes of spin-echo echo-planar images acquired using standard parameters (TR = 3,000 ms, TE = 30 ms, 3-mm slice thickness, 50 contiguous axial sections, refocusing pulse = 89°, FOV = 192 mm, matrix = 64 × 64, acquisition time = 7 min). High-resolution three-dimensional T1-weighted (T1-3D) magnetization-prepared radio-frequency pulses and rapid gradient-echo sampling images were acquired using standard parameters (TR = 1,900 ms, TE = 2.93 ms, 176 contiguous sagittal sections, 1 mm slice thickness, flip angle = 9°, FOV = 260 mm, matrix = 256 × 256). Dual turbo spin-echo sequences were also acquired to exclude patients with brain alterations due to concomitant diseases. CBS is an asymmetric neurological disorder with a prominent involvement of one hemisphere. In our sample, six CBS patients had a prominent involvement of the left hemisphere and five of the right hemisphere. For asymmetry correction purposes, images were flipped in these patients to align the most affected hemisphere with the left side, as previously described elsewhere (10, 23).

### Data Analysis

#### Resting-State fMRI Processing

The data analysis was performed using FMRIB Software Library (FSL package).^1^ The preprocessing steps included brain extraction from T1-3D images using brain extraction software (24), head motion correction, slice timing correction, and spatial smoothening at full width half maximum of 5 mm (14). Further processing was performed using FMRIB’s Non-linear Registration Tool: the functional images were registered linearly with the processed brain extracted images and then were co-registered non-linearly with Montreal Neurological Institute standard space to perform the group comparisons. Following preprocessing, multivariate group probability ICA based on FSL Melodic software was used to perform a temporal concatenation of the spatial ICA maps across all the subjects (25). A high pass temporal filtering cutoff of 100 s was applied. For the stats, we used variance-normalize time courses and automatic dimensionality estimation using temporal concatenated ICA. For the post-stats, we used threshold IC maps with background images to mean high-resolution images. A total of 25 components were obtained from 47 subjects (26). The RSNs were selected visually by careful inspection (27). The following 13 components, representing well-known RSNs, were considered further for dual regression^2^: the default mode, dorsal attention, sensorimotor, executive-control, lateral visual, insula, right and left frontoparietal, salience, auditory, orbitofrontal, cerebellum, and basal ganglia RSNs. Dual regression analysis was adopted to perform a voxel-wise comparison of group ICA by regressing the group ICA maps to an individual set of time series components and re-regressing them back into the subject spatial maps (28). The subject-specific spatial maps were used for the statistical analysis of within and between-network rsFC.

#### Motion Analysis

Head motion may substantially affect rsFC differences between individuals in the same population (29). We performed motion parameter analysis to assess differences between rsFC (17). Absolute and relative displacement values were obtained by using the McFLIRT tool in FSL and assessed across the groups by two-sample *t*-test.

#### Within-Network rsFC

Subject-specific spatial maps of the PSP, CBS, and HS were obtained from the dual regression analysis and were further compared. The spatial maps per component were analyzed by means of a two-sample unpaired *t*-test, entering covariates of no interest (age, sex, and total intracranial volume) into the model. Statistical differences were assessed by randomizing different components per subject using non-parametric permutations, incorporating a threshold-free cluster enhancement technique, and by performing 5,000 random permutations (30). For each RSN (*N* = 13), we compared each patient group with the control group and the two patient groups between them by applying unpaired *t*-test. Brain areas involved in abnormal RSNs were identified on the basis of the Harvard-Oxford atlas (31). Possible correlations between within-network rsFC and clinical scores in patients were assessed by using a general linear model implemented in FSL. The statistical threshold was set at *p* < 0.05, family-wise error (FWE) corrected.

#### Between-Network rsFC

We compared PSP patients, CBS patients, and HS using the FSLNets toolbox^3^ on the MATLAB platform. After normalization of the extracted time courses of all the RSNs identified in each subject, full and partial correlation matrices were calculated between the 13 RSNs. Between-group comparisons of time series correlations were performed using the two-sample unpaired *t*-test. The relationship between clinical scores and between-network rsFC was assessed using Spearman’s rank correlation. The statistical significance threshold was set at *p* < 0.05, FWE corrected.

## Results

The demographic and clinical characteristics of the subjects included in the study are summarized in Table 1. There was no significant difference in age or sex distribution between PSP patients, CBS patients, and HS. The mean UPDRS-III and H&Y clinical scores were similar in the two patient groups. MMSE scores were significantly lower in both patient groups than in HS; MMSE and FAB were significantly lower in PSP than in CBS patients.

**Table 1:** Demographic and clinical characteristics in patients with progressive supranuclear palsy (PSP), corticobasal syndrome (CBS), and healthy controls.

	Healthy controls (*n* = 16)	Patients with PSP (*n* = 20)	Patients with CBS (*n* = 11)	*p* Value*	*p* Value**	*p* Value***
Age (years)	69.38 ± 4.9	69.28 ± 4.8	66 ± 3.9	0.95	0.06	0.06
Male/female	7/9	10/10	3/8	0.97	0.38	0.21
Disease duration (years)	–	3.17 ± 1.75	2.72 ± 1.27	–	–	0.46
UPDRS-III	–	27.2 ± 16.4	33.9 ± 24.5	–	–	0.36
H&Y	–	3.05 ± 1.02	2.3 ± 1.7	–	–	0.13
MMSE	29.4 ± 0.9	24.06 ± 3.78	27.45 ± 2	**0.004**	**0.002**	**0.01**
FAB	–	11.05 ± 3.64	15.7 ± 1.55	–	–	**0.0004**

*UPDRS-III, Unified Parkinson’s Disease Rating Scale; H&Y, Hoehn and Yahr scale; MMSE, Mini-Mental State Examination; FAB, Frontal Assessment Battery*.

*Values are reported as mean ± SD*.

*Differences between healthy controls and PSP (unpaired *t*-test).

**Differences between healthy controls and CBS (unpaired *t*-test).

***Differences between PSP and CBS (unpaired *t*-test).

*Sex differences were assessed by χ^2^ test*.

*Significant p values are indicated in bold*.

Maximum absolute head motion was 1.38 mm in PSP, 2.98 mm in CBS, and 1.20 mm in HS. Mean absolute and relative displacement values were 0.69 ± 0.30 and 0.07 ± 0.03 mm in PSP, 0.75 ± 0.69 and 0.10 ± 0.10 mm in CBS, and 0.40 ± 0.28 and 0.07 ± 0.06 mm in HS. There were no significant differences in motion parameters between any of the three groups of subjects.

### Within-Network Connectivity

**Figure 1:**
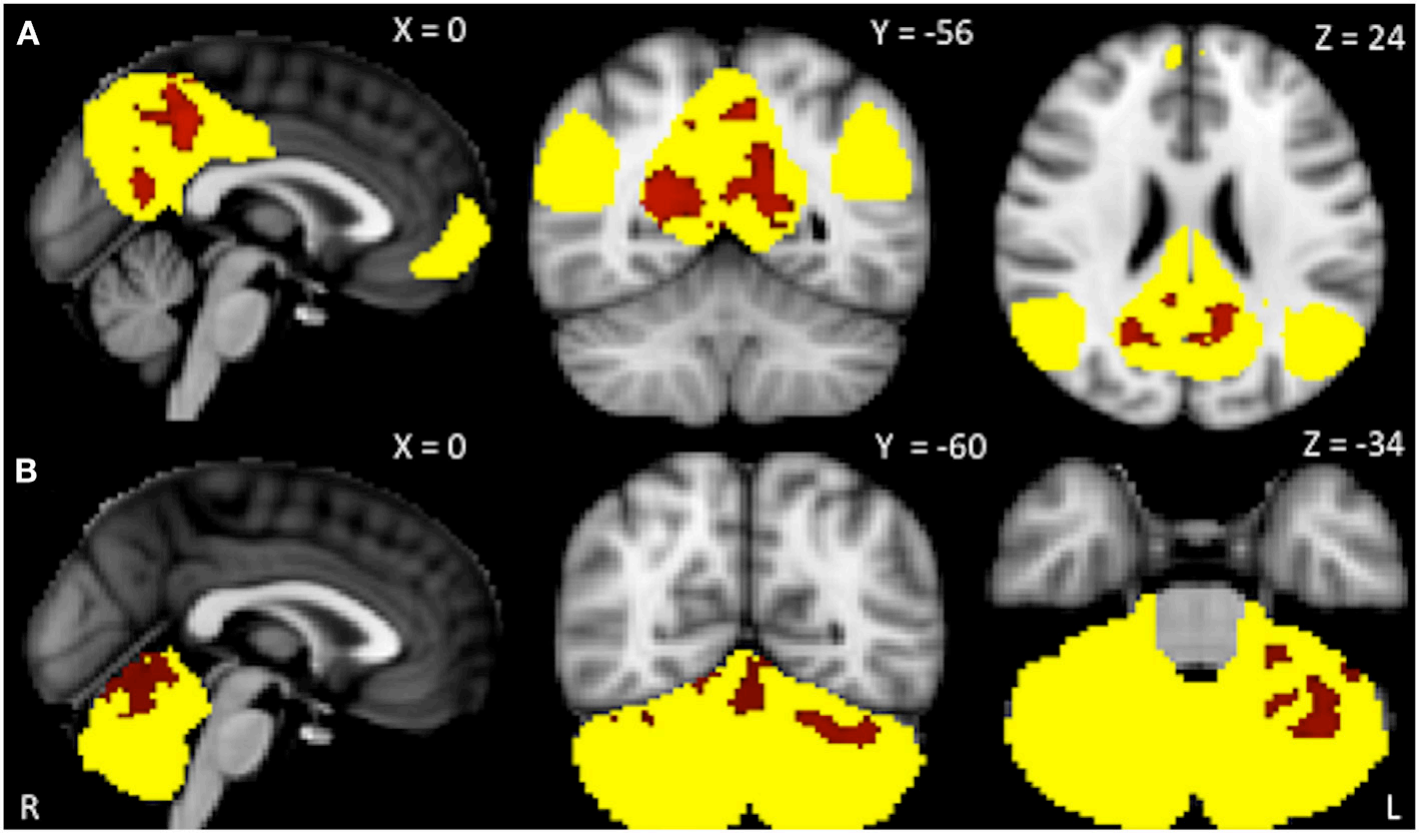
Resting-state networks showing significantly higher within-network resting-state functional connectivity (rsFC) in PSP than in healthy subjects (*yellow*). *Red* areas represent significantly increased rsFC. **(A)** Default mode network: precuneus and posterior cingulate cortex bilaterally; **(B)** cerebellum network: left crus I, I–VI lobule, I–V lobule, and vermis. Data are shown at *p* < 0.05, corrected for family-wise error.

**Figure 2:**
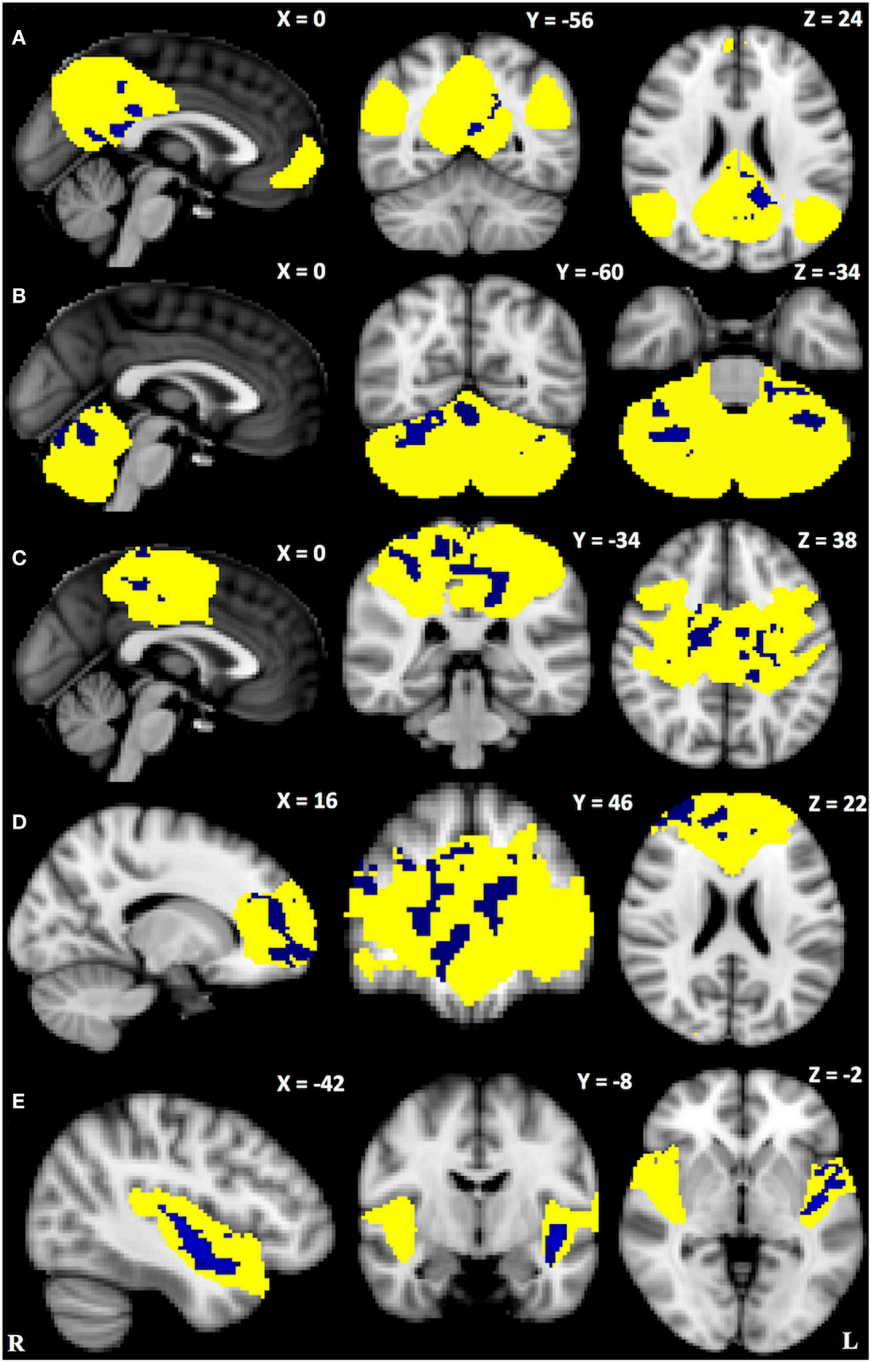
Resting-state networks showing significantly higher within-network connectivity in corticobasal syndrome than in healthy subjects (*yellow*). *Blue* areas represent significantly increased resting-state functional connectivity. **(A)** Default mode network: precuneus, posterior cingulate cortex and lingual gyrus bilaterally; **(B)** cerebellum network: crus I bilaterally, left I–VI lobule, right I–VI lobule, and vermis; **(C)** sensorimotor network: pre- and postcentral gryus, sensorimotor area, posterior cingulate cortex, superior parietal lobule, and supramarginal gyrus bilaterally; **(D)** executive-control network: frontal pole, superior frontal gyrus, paracingulate gyrus, anterior cingulate cortex bilaterally as well as right middle frontal gyrus; **(E)** left insular cortex: left planum polare and anterior superior temporal gyrus. Data are shown at *p* < 0.05, corrected for family-wise error.

**Figure 3:**
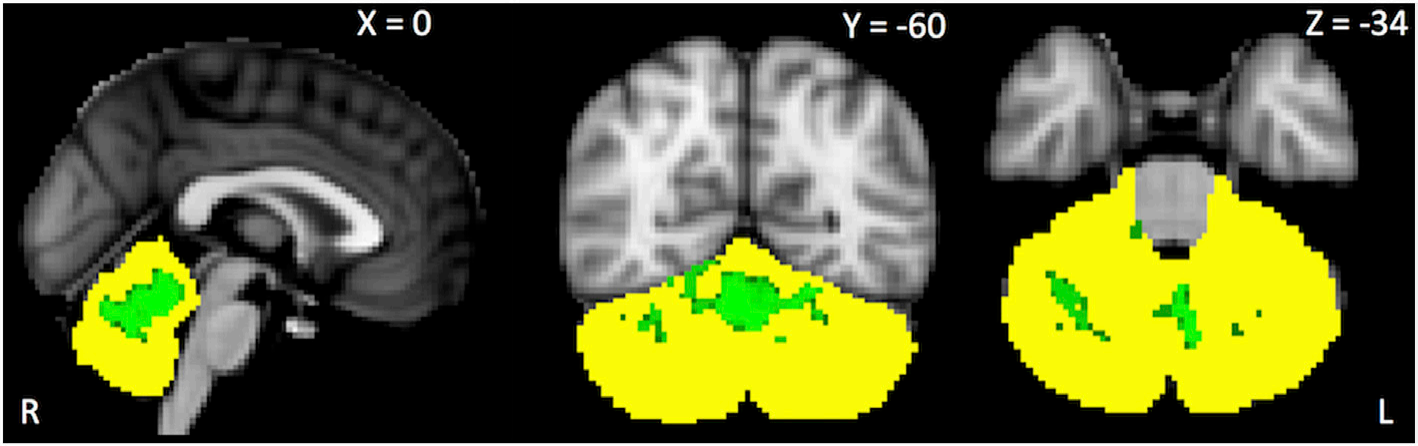
*Green* areas represent areas of positive correlations between the Mini-Mental State Evaluation scores and within-network cerebellar resting-state functional connectivity. Results are shown at *p* < 0.05, corrected for family-wise error.

Within-network rsFC was higher in both PSP and CBS than in HS in the default mode and cerebellum RSNs (Figures 1 and 2A,B). In addition, within-network rsFC in sensorimotor, executive-control and insula RSNs was higher in CBS patients than in HS (Figures 2C–E). When PSP and CBS patients were compared, we did not observe any significant difference in within-network rsFC. The MMSE score positively correlated with cerebellar within-network rsFC in the left crus I, left I–VI lobule, right I–V lobule, and vermis in PSP patients (Figure 3). No other significant correlations between the clinical scores and within-network rsFC were observed.

### Between-Network Connectivity

**Figure 4:**
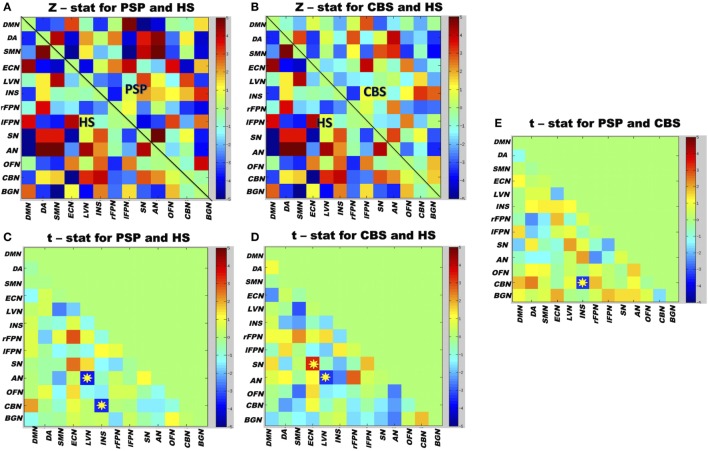
Matrix map of partial correlation obtained from 13 resting-state networks (RSNs) time courses of whole group of participants. **(A)** The between-network partial correlation maps in PSP and healthy subjects (HS). **(B)** The between-network partial correlation maps in corticobasal syndrome (CBS) and HS. **(C,D)** The *t*-test values when PSP and CBS were compared separately with HS. **(E)** The *t*-test values when both the patient groups were compared with each other. Yellow stars represent significant differences in between-network connectivity after group comparisons; color bars indicate the *t*-stat values. Red boxes represent increased between-network resting-state functional connectivity (rsFC), and blue boxes represent decreased between-network rsFC. The 13 RSNs: DMN, default mode network; DA, dorsal attention; SMN, sensorimotor network; ECN, executive-control network; LVN, lateral visual network; INS, insula; rFPN, right fronto-parietal network; lFPN, left fronto-parietal network; SN, salience network; AN, auditory network; OFN, orbitofrontal network; CBN, cerebellum network; BGN, basal ganglia network. Results are shown at *p* < 0.05, corrected for family-wise error.

Both PSP and CBS patients exhibited lower rsFC between the lateral visual and auditory RSNs than HS. PSP patients exhibited lower rsFC between the cerebellum and insula RSNs than CBS and HS. Finally, CBS patients exhibited higher rsFC between the salience and executive-control RSNs than HS (Figure 4). No significant correlations between the clinical scores and between-network rsFC were observed.

## Discussion

In the present study, we used an ICA-based method to evaluate rsFC changes in PSP and CBS patients. The analysis of within- and between-network rsFC revealed functional abnormalities involving several RSNs in both conditions.

A novel finding of the present study is the increased within-network rsFC in the default mode and cerebellum RSNs observed in PSP and CBS patients, which may reflect widespread neurodegenerative phenomena in both conditions. Since the default mode network is mainly associated with cognitive functions (32, 33), the increased within-network rsFC observed in both PSP and CBS may underlie the cognitive impairment often observed in these conditions (34). The correlation found in patients with PSP between the within-network rsFC of the cerebellum and MMSE scores provides further insight into the possible mechanisms underlying the cognitive impairment in this condition. The lack of a similar correlation in CBS may either be due to the lower cognitive decline we observed in these patients than in PSP or indicate different underlying pathophysiological mechanisms. The cerebellum contributes to several motor and non-motor functions (35), and the significance of increased rsFC within the cerebellum RSN in PSP and CBS has yet to be fully clarified.

We observed increased within-network rsFC in the sensorimotor RSN, which may reflect tau burden and gray matter loss in the peri-rolandic cortex, in CBS patients alone (36, 37). This finding is consistent with previous studies showing an increased involvement of cortical areas in CBS (5, 38). Accordingly, we observed increased within-network rsFC in the executive-control and insula RSNs. Sensorimotor RSNs play a predominant role in motor planning, preparation, and execution (39), while the executive-control and insula RSNs are prevalently associated with cognitive, attentional, and emotional processes (40, 41). The rsFC abnormalities we observed may contribute to various cortical symptoms in CBS, e.g., alien limb, myoclonus, and cortical sensory loss (42).

Both PSP and CBS patients displayed decreased rsFC between the lateral visual and auditory RSNs, which are anatomically interconnected (43) and are involved in high order multisensory integration (39, 44). The impaired functional coupling between the lateral visual and auditory RSNs might disrupt multimodal sensory input processing, although the clinical correlate of this abnormality has yet to be clarified.

PSP patients also displayed lower rsFC between the cerebellum and insula RSNs than either CBS patients or HS, a finding likely to be due to altered structural connections between the cerebellum and limbic system (45). As the cerebellum and insula are both associated with emotional processing (46, 47), a lower functional interaction may be the underlying substrate of the emotional changes and altered emotional processing often reported in PSP (48). Finally, we observed increased rsFC between the salience and executive-control RSNs in patients with CBS, but not in those with PSP. These two RSNs are interconnected and play a role in cognition (9, 49). In particular, the salience RSN plays an important role in cognitive control by integrating input stimuli and identifying the most relevant ones (50), whereas the executive-control RSN is involved in working memory, judgment, decision-making, and goal-directed behavior (51, 52). The increased functional connections between these networks may be interpreted as a compensatory plastic rearrangement aimed at preserving cognitive function (53).

The increased within-network rsFC we observed in PSP and CBS is consistent with the results of previous studies that reported enhanced functional connectivity in other diseases, i.e., frontotemporal dementia, Parkinson’s disease, and Huntington’s disease (15–17). Taken together, these results point to higher synchronous baseline activity in various RSNs induced by neurodegeneration. The resulting loss of network specialization (17, 54) and/or inhibitory interneurons (55, 56) may contribute to a generalized spread of neuronal activity, abnormal oscillations, and synchronization (57). Alternatively, the increase in functional connectivity may represent a plasticity-related shift in neuronal activity from atrophic to intact brain structures, as has been suggested by other authors (15). Between-network rsFC changes have also been reported in other neurodegenerative conditions. Reduced between-network rsFC is believed to reflect the structural decoupling of interregional networks resulting from degeneration of long-range white matter fibers, whereas increased between-network rsFC is believed to reflect a compensatory or maladaptive plastic rearrangement of coupling between interregional networks (53).

This study presents a number of strengths as well as some limitations. We used an unbiased approach of whole-brain rsFC analysis that provides an overall view of spontaneous brain activity at rest, which is arranged in clearly defined functional networks (14). Previous rsFC investigations on PSP, which, unlike ours, used an *a priori*-based approach, reported reduced rsFC of pre-defined regions of interest, i.e., between subcortical nuclei and cortical and cerebellar areas (10–12). Results obtained by means of different fMRI postprocessing methodologies and including heterogeneous samples of patients cannot, however, easily be compared. Since PSP and CBS are rare neurodegenerative disorders, it is often difficult to recruit large samples of patients. To avoid loss of information (statistical error type II), we used a less conservative approach of analysis, correcting the results for multiple comparisons at a voxel level but not at an RSN level. Moreover, we only recruited patients with a probable clinical diagnosis since a definite diagnosis of the disease can only be made by performing a postmortem examination.

## Conclusion

In conclusion, this study provides novel information on abnormal functional connectivity in PSP and CBS. The increased within-network rsFC in well-defined functional networks is likely to be due to disturbed neuronal activity caused by neurodegeneration. Changes in between-network rsFC reflect structural disconnection due to white matter fiber damage (6) or an attempt at functional reorganization in response to neurological disruption (53). The results of this study provide a better understanding of the pathophysiology of PSP and CBD. Future studies are warranted to further clarify the role of altered brain connectivity and its relationship with motor and non-motor features in these conditions.

## Ethics Statement

The study approved by our institutional review board and conformed to the declaration of Helsinki. All participants gave their written informed consent.

## Author Contributions

All the authors have contributed equally.

## Conflict of Interest Statement

On behalf of all the authors, the corresponding author states that there is no conflict of interest.

## Footnotes

^1^http://www.fmrib.ox.ac.uk/fsl.

^2^https://fsl.fmrib.ox.ac.uk/fsl/fslwiki/DualRegression.

^3^http://fsl.fmrib.ox.ac.uk/fsl/fslwiki/FSLNets.

## Abbreviations

CBS, corticobasal syndrome; FAB, Frontal Assessment Battery; FWE, family-wise error; H&Y, Hoehn and Yahr; HS, healthy subjects; ICA, independent component analysis; MMSE, Mini-Mental State Evaluation; PSP, progressive supranuclear palsy; rsFC, resting-state functional connectivity; rsfMRI, resting-state functional magnetic resonance imaging; RSNs, resting-state networks; UPDRS, Unified Parkinson’s Disease Rating Scale.

## References

[1] Williams DR Characteristics of two distinct clinical phenotypes in pathologically proven progressive supranuclear palsy: Richardson’s syndrome and PSP-parkinsonism. Brain (2005) 128:1247–58. 10.1093/brain/awh488 15788542

[2] Armstrong MJ Litvan I Lang AE Bak TH Bhatia KP Borroni B Criteria for the diagnosis of corticobasal degeneration. Neurology (2013) 80:496–503. 10.1212/WNL.0b013e31827f0fd1 23359374 PMC3590050

[3] Josephs KA Whitwell JL Dickson DW Boeve BF Knopman DS Petersen RC Voxel-based morphometry in autopsy proven PSP and CBD. Neurobiol Aging (2008) 29:280–9. 10.1016/j.neurobiolaging.2006.09.019 17097770 PMC2702857

[4] Piattella MC Upadhyay N Bologna M Sbardella E Tona F Formica A Neuroimaging evidence of gray and white matter damage and clinical correlates in progressive supranuclear palsy. J Neurol (2015) 262:1850–8. 10.1007/s00415-015-7779-3 25980906

[5] Upadhyay N Suppa A Piattella MC Di Stasio F Petsas N Colonnese C Gray and white matter structural changes in corticobasal syndrome. Neurobiol Aging (2016) 37:82–90. 10.1016/j.neurobiolaging.2015.10.011 26545629

[6] Upadhyay N Suppa A Piattella MC Bologna M Di Stasio F Formica A MRI gray and white matter measures in progressive supranuclear palsy and corticobasal syndrome. J Neurol (2016) 263(10):2022–31. 10.1007/s00415-016-8224-y 27411806

[7] Whitwell JL Schwarz CG Reid RI Kantarci K Jack CR Josephs KA. Diffusion tensor imaging comparison of progressive supranuclear palsy and corticobasal syndromes. Parkinsonism Relat Disord (2014) 20:493–8. 10.1016/j.parkreldis.2014.01.023 24656943

[8] Agosta F Galantucci S Svetel M Lukić MJ Copetti M Davidovic K Clinical, cognitive, and behavioural correlates of white matter damage in progressive supranuclear palsy. J Neurol (2014) 261:913–24. 10.1007/s00415-014-7301-3 24599641

[9] Fox MD Raichle ME Spontaneous fluctuations in brain activity observed with functional magnetic resonance imaging. Nat Rev Neurosci (2007) 8:700–11. 10.1038/nrn2201 17704812

[10] Whitwell JL Avula R Master A Vemuri P Senjem ML Jones DT Disrupted thalamocortical connectivity in PSP: a resting-state fMRI, DTI, and VBM study. Parkinsonism Relat Disord (2011) 17:599–605. 10.1016/j.parkreldis.2011.05.013 21665514 PMC3168952

[11] Gardner RC Boxer AL Trujillo A Mirsky JB Guo CC Gennatas ED Intrinsic connectivity network disruption in progressive supranuclear palsy: network disruption in PSP. Ann Neurol (2013) 73:603–16. 10.1002/ana.23844 23536287 PMC3732833

[12] Piattella MC Tona F Bologna M Sbardella E Formica A Petsas N Disrupted resting-state functional connectivity in progressive supranuclear palsy. AJNR Am J Neuroradiol (2015) 36:915–21. 10.3174/ajnr.A4229 25655870 PMC7990581

[13] Seeley WW Crawford RK Zhou J Miller BL Greicius MD. Neurodegenerative diseases target large-scale human brain networks. Neuron (2009) 62:42–52. 10.1016/j.neuron.2009.03.024 19376066 PMC2691647

[14] Beckmann CF DeLuca M Devlin JT Smith SM. Investigations into resting-state connectivity using independent component analysis. Philos Trans R Soc Lond B Biol Sci (2005) 360:1001–13. 10.1098/rstb.2005.1634 16087444 PMC1854918

[15] Rytty R Nikkinen J Paavola L Abou Elseoud A Moilanen V Visuri A GroupICA dual regression analysis of resting state networks in a behavioral variant of frontotemporal dementia. Front Hum Neurosci (2013) 7:461. 10.3389/fnhum.2013.00461 23986673 PMC3752460

[16] Onu M Badea L Roceanu A Tivarus M Bajenaru O Increased connectivity between sensorimotor and attentional areas in Parkinson’s disease. Neuroradiology (2015) 57:957–68. 10.1007/s00234-015-1556-y 26174425

[17] Werner CJ Dogan I Saß C Mirzazade S Schiefer J Shah NJ Altered resting-state connectivity in Huntington’s disease. Hum Brain Mapp (2014) 35:2582–93. 10.1002/hbm.22351 23982979 PMC6869508

[18] Litvan I Agid Y Calne D Campbell G Dubois B Duvoisin RC Clinical research criteria for the diagnosis of progressive supranuclear palsy (Steele-Richardson-Olszewski syndrome): report of the NINDS-SPSP international workshop. Neurology (1996) 47:1–9. 10.1212/WNL.47.1.1 8710059

[19] Angelo Antonini GA Validation of the Italian version of the movement disorder society – Unified Parkinson’s Disease Rating Scale. Neurol Sci (2012) 34:683–7. 10.1007/s10072-012-1112-z 22678179

[20] Hoehn MM Yahr MD Parkinsonism: onset, progression, and mortality. Neurology (1998) 50:318 10.1212/WNL.50.2.318 9484345

[21] Folstein MF Folstein SE McHugh PR “Mini-mental state”: a practical method for grading the cognitive state of patients for the clinician. J Psychiatr Res (1975) 12:189–98. 10.1016/0022-3956(75)90026-6 1202204

[22] Dubois B Slachevsky A Litvan I Pillon B. The FAB: a Frontal Assessment Battery at bedside. Neurology (2000) 55:1621–6. 10.1212/WNL.55.11.1621 11113214

[23] Upadhyay N Suppa A Piattella MC Di Stasio F Petsas N Colonnese C Corrigendum to “Gray and white matter structural changes in corticobasal syndrome” [Neurobiol. Aging 37 (2016) 82–90]. Neurobiol Aging (2017) 53:199 10.1016/j.neurobiolaging.2017.03.019 26545629

[24] Smith SM. Fast robust automated brain extraction. Hum Brain Mapp (2002) 17:143–55. 10.1002/hbm.10062 12391568 PMC6871816

[25] Beckmann CF Smith SM. Probabilistic independent component analysis for functional magnetic resonance imaging. IEEE Trans Med Imaging (2004) 23:137–52. 10.1109/TMI.2003.822821 14964560

[26] Beckmann CF Smith SM. Tensorial extensions of independent component analysis for multisubject FMRI analysis. Neuroimage (2005) 25:294–311. 10.1016/j.neuroimage.2004.10.043 15734364

[27] Rosazza C Minati L Resting-state brain networks: literature review and clinical applications. Neurol Sci (2011) 32:773–85. 10.1007/s10072-011-0636-y 21667095

[28] Beckmann CF Mackay CE Filippini N Smith SM Group comparison of resting-state FMRI data using multi-subject ICA and dual regression. Neuroimage (2009) 47:S148 10.1016/S1053-8119(09)71511-3

[29] Van Dijk KRA Sabuncu MR Buckner RL. The influence of head motion on intrinsic functional connectivity MRI. Neuroimage (2012) 59:431–8. 10.1016/j.neuroimage.2011.07.044 21810475 PMC3683830

[30] Nichols TE Holmes AP. Nonparametric permutation tests for functional neuroimaging: a primer with examples. Hum Brain Mapp (2002) 15:1–25. 10.1002/hbm.1058 11747097 PMC6871862

[31] Desikan RS Ségonne F Fischl B Quinn BT Dickerson BC Blacker D An automated labeling system for subdividing the human cerebral cortex on MRI scans into gyral based regions of interest. Neuroimage (2006) 31:968–80. 10.1016/j.neuroimage.2006.01.021 16530430

[32] Greicius MD Krasnow B Reiss AL Menon V. Functional connectivity in the resting brain: a network analysis of the default mode hypothesis. Proc Natl Acad Sci U S A (2003) 100:253–8. 10.1073/pnas.0135058100 12506194 PMC140943

[33] Raichle ME Snyder AZ. A default mode of brain function: a brief history of an evolving idea. Neuroimage (2007) 37:1083–90. 10.1016/j.neuroimage.2007.02.041 17719799

[34] Greicius MD Srivastava G Reiss AL Menon V Default-mode network activity distinguishes Alzheimer’s disease from healthy aging: evidence from functional MRI. Proc Natl Acad Sci U S A (2004) 101:4637–42. 10.1073/pnas.0308627101 15070770 PMC384799

[35] Koziol LF Budding D Andreasen N D’Arrigo S Bulgheroni S Imamizu H Consensus paper: the cerebellum’s role in movement and cognition. Cerebellum (2014) 13:151–77. 10.1007/s12311-013-0511-x 23996631 PMC4089997

[36] Dickson DW Bergeron C Chin SS Duyckaerts C Horoupian D Ikeda K Office of rare diseases neuropathologic criteria for corticobasal degeneration. J Neuropathol Exp Neurol (2002) 61:935–46. 10.1093/jnen/61.11.935 12430710

[37] Lee SE Rabinovici GD Mayo MC Wilson SM Seeley WW DeArmond SJ Clinicopathological correlations in corticobasal degeneration. Ann Neurol (2011) 70:327–40. 10.1002/ana.22424 21823158 PMC3154081

[38] Dutt S Binney RJ Heuer HW Luong P Attygalle S Bhatt P Progression of brain atrophy in PSP and CBS over 6 months and 1 year. Neurology (2016) 87:2016–25. 10.1212/WNL.0000000000003305 27742814 PMC5109951

[39] Smith SM Fox PT Miller KL Glahn DC Fox PM Mackay CE Correspondence of the brain’s functional architecture during activation and rest. Proc Natl Acad Sci U S A (2009) 106:13040–5. 10.1073/pnas.0905267106 19620724 PMC2722273

[40] Menon V Uddin LQ. Saliency, switching, attention and control: a network model of insula function. Brain Struct Funct (2010) 214:655–67. 10.1007/s00429-010-0262-0 20512370 PMC2899886

[41] Beaty RE Benedek M Kaufman SB Silvia PJ. Default and executive network coupling supports creative idea production. Sci Rep (2015) 5:10964. 10.1038/srep10964 26084037 PMC4472024

[42] Kouri N Murray ME Hassan A Rademakers R Uitti RJ Boeve BF Neuropathological features of corticobasal degeneration presenting as corticobasal syndrome or Richardson syndrome. Brain (2011) 134:3264–75. 10.1093/brain/awr234 21933807 PMC3212714

[43] Falchier A Schroeder CE Hackett TA Lakatos P Nascimento-Silva S Ulbert I Projection from visual areas V2 and prostriata to caudal auditory cortex in the monkey. Cereb Cortex (2010) 20:1529–38. 10.1093/cercor/bhp213 19875677 PMC2882821

[44] Budinger E Heil P Hess A Scheich H. Multisensory processing via early cortical stages: connections of the primary auditory cortical field with other sensory systems. Neuroscience (2006) 143:1065–83. 10.1016/j.neuroscience.2006.08.035 17027173

[45] Schutter DJLG van Honk J The cerebellum on the rise in human emotion. Cerebellum (2005) 4:290–4. 10.1080/14734220500348584 16321885

[46] Liu Z Xu C Xu Y Wang Y Zhao B Lv Y Decreased regional homogeneity in insula and cerebellum: a resting-state fMRI study in patients with major depression and subjects at high risk for major depression. Psychiatry Res (2010) 182:211–5. 10.1016/j.pscychresns.2010.03.004 20493670

[47] Adamaszek M D’Agata F Ferrucci R Habas C Keulen S Kirkby KC Consensus paper: cerebellum and emotion. Cerebellum (2017) 16(2):552–76. 10.1007/s12311-016-0815-8 27485952

[48] Ghosh BCP Rowe JB Calder AJ Hodges JR Bak TH. Emotion recognition in progressive supranuclear palsy. J Neurol Neurosurg Psychiatry (2009) 80:1143–5. 10.1136/jnnp.2008.155846 19762901 PMC3044450

[49] Young CB Raz G Everaerd D Beckmann CF Tendolkar I Hendler T Dynamic shifts in large-scale brain network balance as a function of arousal. J Neurosci (2017) 37:281–90. 10.1523/JNEUROSCI.1759-16.2017 28077708 PMC6596574

[50] Seeley WW Menon V Schatzberg AF Keller J Glover GH Kenna H Dissociable intrinsic connectivity networks for salience processing and executive control. J Neurosci (2007) 27:2349–56. 10.1523/JNEUROSCI.5587-06.2007 17329432 PMC2680293

[51] Koechlin E Summerfield C. An information theoretical approach to prefrontal executive function. Trends Cogn Sci (2007) 11:229–35. 10.1016/j.tics.2007.04.005 17475536

[52] Burrell JR Hodges JR Rowe JB Cognition in corticobasal syndrome and progressive supranuclear palsy: a review. Mov Disord (2014) 29:684–93. 10.1002/mds.25872 24757116

[53] Hillary FG Roman CA Venkatesan U Rajtmajer SM Bajo R Castellanos ND. Hyperconnectivity is a fundamental response to neurological disruption. Neuropsychology (2015) 29:59–75. 10.1037/neu0000110 24933491

[54] Rajah MN D’Esposito M. Region-specific changes in prefrontal function with age: a review of PET and fMRI studies on working and episodic memory. Brain (2005) 128:1964–83. 10.1093/brain/awh608 16049041

[55] Halliday G. Clinicopathological aspects of motor parkinsonism. Parkinsonism Relat Disord (2007) 13(Suppl 3):S208–10. 10.1016/S1353-8020(08)70003-8 18267237

[56] Conte A Khan N Defazio G Rothwell JC Berardelli A Pathophysiology of somatosensory abnormalities in Parkinson disease. Nat Rev Neurol (2013) 9:687–97. 10.1038/nrneurol.2013.224 24217516

[57] Galvan A Devergnas A Wichmann T. Alterations in neuronal activity in basal ganglia-thalamocortical circuits in the parkinsonian state. Front Neuroanat (2015) 9:5. 10.3389/fnana.2015.00005 25698937 PMC4318426

